# Lassa hemorrhagic shock syndrome‐on‐a‐chip

**DOI:** 10.1002/bit.27636

**Published:** 2020-12-04

**Authors:** Huaqi Tang, Yasmine Abouleila, Alireza Mashaghi

**Affiliations:** ^1^ Medical Systems Biophysics and Bioengineering, Leiden Academic Centre for Drug Research Leiden University Leiden The Netherlands

**Keywords:** drug, Lassa hemorrhagic fever, microvessel, Organ Chips, vascular permeability

## Abstract

Lack of experimental human models hinders research on Lassa hemorrhagic fever and the development of treatment strategies. Here, we report the first chip‐based model for Lassa hemorrhagic syndrome. The chip features a microvessel interfacing collagen network as a simple mimic for extracellular matrix, allowing for quantitative and real‐time vascular integrity assessment. Luminal infusion of Lassa virus‐like particles led to a dramatic increase in vascular permeability in a viral load‐dependent manner. Using this platform, we showed that Fibrin‐derived peptide FX06 can be used to suppress the vascular integrity loss. This simple chip‐based model proved promising in the assessment of disease severity and provides an easy‐to‐use platform for future investigation of Lassa pathogenesis and drug development in a human‐like setting.

## Graphical abstract

An organ‐on‐a‐chip model that imitates Lassa hemorrhagic shock syndrome was developed and used to test drug candidate. The chip is composed of a microvessel interfacing collagen network as a simple mimic for extracellular matrix, which can recapitulate the physiological barrier function of a natural vessel and luminal infusion of Lassa virus‐like particles led to a dramatic increase in vascular permeability. Using this platform, a potential drug, Fibrin‐derived peptide FX06, shows its ability to suppress the Lassa‐induced vascular integrity loss.

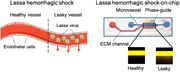

## 1 INTRODUCTION

Lassa virus belongs to the *Arenaviridae* family and causes severe vascular permeability in patients which often is the main cause of death with high mortality rates (Baccala et al., 2014; Wiley et al., 2019). An estimated 100,000–300,000 Lassa infections and approximately 5000 deaths occur annually across West Africa, such numbers impose huge health and socioeconomic burden on the affected countries as well as international organizations (Okokhere et al., 2018; Wiley et al., 2019). The recent (2018–2019) outbreaks in Nigeria featured alarmingly high fatality rates approximately 25% (Sattler et al., 2020). Due to the ease of spread, disease severity, and high fatality, the disease has received widespread attention, in which, WHO classified Lassa virus as a category A pathogen and a bioterrorism threat (Siddle et al., 2018). Although the antiviral drug ribavirin shows effectiveness if given early in the course of disease (Eberhardt et al., 2019) and several human monoclonal antibodies have been investigated using animal models (Mire et al., 2017; Robinson et al., 2016), there are currently no licensed vaccines or targeted drug therapies for the prevention or treatment of Lassa virus disease.

The development of medical countermeasures and early detection of Lassa has been hindered due to the lack of experimental models and sensitive detection tools. Studies with currently available laboratory animal models, for example, mice, guinea pigs, and nonhuman primates (NHPs), have provided valuable information regarding the pathogenesis of Lassa virus infection in general, and not for the hemorrhagic shock (HS) syndrome, which is often the main cause of death (Hallam et al., 2018; Sattler et al., 2020). Additionally, current animal studies lack direct comparisons between responses in patients with severe versus mild infections (Baccala et al., 2014). The use of these animals is limited by the paucity of reagents and genetic information, the need for BSL‐4 facilities, and associated ethical issues. More specifically, animal models cannot fully recapitulate the physiology and pathology of human organs (Mestas & Hughes, 2004), which further hinder the necessary research on Lassa virus disease required for vaccination and targeted drug development.

Organ Chips provide a pivotal opportunity to overcome the previously mentioned limitations associated with animal models. Organ Chips are engineered microfluidic devices that can replicate the complex structures and physiological functions of major functional units of human organs and mimic human organ‐specific responses (Bhatia & Ingber, 2014). Chip‐based disease models are thus becoming important research tools in numerous applications, examples include but are not limited to, diabetic nephropathy and drug toxicities on a Kidney Glomerulus Chip (Musah et al., 2017), intravascular thrombosis on a Lung Alveolus Chip (Jain et al., 2018), Alzheimer's disease on a Brain Chip (Park et al., 2015), and radiation and drug toxicities (Junaid et al., 2017), as well as modeling a rare genetic disease on a Bone Marrow Chip (Chou et al., 2020). Only recently the technology has been introduced to the field of virology, with special focus on emerging fatal diseases such as Ebola and SARS‐CoV‐2 (Tang et al., 2020). However, no chip‐based model of Lassa HS has been introduced as yet.

In this communication, we present the first chip‐based model for Lassa HS. The model includes a perfusable and endothelialized microvasculature‐on‐a‐chip featuring a collagen hydrogel that minimally mimics the extracellular matrix (ECM) of host tissues. Luminal infusion of Lassa virus‐like particles (VLPs) leads to leakage of fluorescently labeled albumin from the engineered vessels, the resultant impairment of vascular integrity can be readily quantified. In addition, we investigated whether Fibrin‐derived peptide FX06 is able to suppress the Lassa‐induced vascular integrity loss and can be a promising drug for Lassa HS. Our proof‐of‐concept study provides a low‐cost, easy‐to‐use and high‐throughput platform that will not only be useful for studying the pathogenesis of Lassa in a human‐like setting but also be critical for diagnostics and drug development.

**Figure 1:**
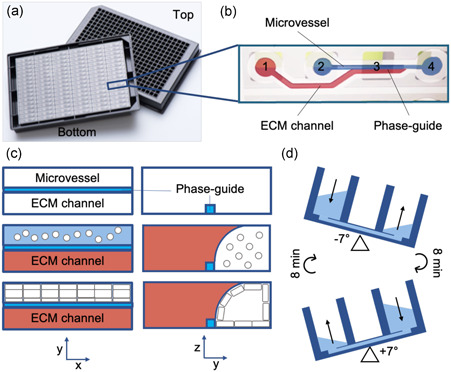
Lassa HS‐on‐a‐Chip. (a) Schematic diagram of the OrganoPlate used for perfusable microvessel culture, based on a 384 well plate interface on top and 96 microfluidic devices integrated in the bottom. (b) Each microfluidic device consists of a microvessel and an ECM channel separated by a phase‐guide, which prevents the gel from flowing into the adjacent channels. Every microfluidic structure is positioned underneath four adjacent wells; gel inlet (1), medium inlet (2), imaging/observation window (3), and medium outlet (4). (c) Method for microvasculature culture. Collagen gel is seeded into the ECM channel and polymerized, then HUVECs suspension is seeded into the perfusion channel. The perfusion is started by placing the plate on a tilted rocking platform with a rocking interval of 8 min, which creates a height difference resulting in gravity driven, continuous, and bidirectional perfusion flow. (d) HUVECs will then grow as a confluent monolayer against the collagen gel and channel walls, resulting in a microvessel with a perfusable lumen. ECM, extracellular matrix; HS, hemorrhagic shock; HUVEC, human umbilical vein endothelial cell [Color figure can be viewed at wileyonlinelibrary.com]

To provide the required proof‐of‐principle for this approach and to ensure that the platform can be extended to a low‐cost, easy‐to‐use and high‐throughput platform suitable for diagnostics, we included the minimal components needed to model the process. A total of 96 perfusable and endothelialized microvessels within a fabricated phase‐guide channel system was cultured with primary human umbilical vein endothelial cells (HUVECs) at the interface of a collagen type I network and subjected to continuous perfusion. The phase‐guide design allows the interfacing of two parallel channels without any physical wall in‐between, while maintaining the ability to grow tissues or form ECM without mixing the channels’ contents (Figure 1) (Vulto et al., 2011). To further ensure that these engineered vessels recapitulated the vascular barrier function of a natural vessel in vivo, we measured the transport of fluorescence‐labeled albumin across the endothelial wall into the collagen network. Our previous work has demonstrated that the engineered vessel is impermeable but sensitive to physiological vasoactive stimuli. Upon exposure to histamine, an endogenous biogenic amine that can induce vascular permeability during inflammatory processes, increase in the vascular permeability was observed (Junaid et al., 2020).

**Figure 2:**
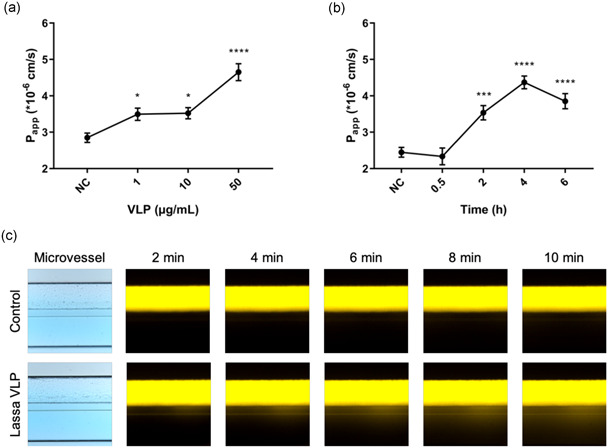
Lassa VLPs induced vascular permeability increase in the Lassa HS chip platform. (a) Apparent permeability (*P*_app_) of microvessels when exposed to Lassa VLPs for 4 h with the indicated concentrations (1, 10, 50 μg/ml). (b) *P*_app_ of microvessels in response to 50 μg/ml Lassa VLPs at different time points (0.5, 2, 4, 6 h). (c) Time‐lapse fluorescence images of albumin perfusing in the negative control microvessel channel and diffusing from the Lassa VLPs treated microvessel to the ECM channel (*n* = 12). HS, hemorrhagic shock; VLP, virus‐like particle [Color figure can be viewed at wileyonlinelibrary.com]

To investigate whether Lassa HS can be disentangled from Lassa virus infection, various concentrations of Lassa VLPs were infused into the engineered vessels to determine whether Lassa VLPs alone are sufficient to induce permeability and whether the extent of permeability is viral load or time dependent. This allowed us to disassemble the complexity of Lassa virus disease and to make a minimal and safe chip system tailored for studying Lassa HS. Infusion of VLPs led to a dramatic increase in the permeability of the engineered microvessels (Figure 2a,b). As shown in Figure 2c, we observed no leakage of albumin from the engineered vessels (control) within a 10 min interval during the permeability assay. Permeability was, however, induced by the administration of Lassa VLPs indicating that the endothelial wall is not passive and responds to stimuli as expected. This platform proved promising in the assessment of disease severity as the in vitro analysis showed a clear correlation between the integrity loss and the viral load. The VLPs used in these experiments were structurally and morphologically similar to native Lassa virus virions but lack replicative functions (Branco et al., 2010), depicting that the viral components interact directly with endothelial cells and affect their barrier function, presumably by affecting cellular mechanics and intercellular interactions.

**Figure 3:**
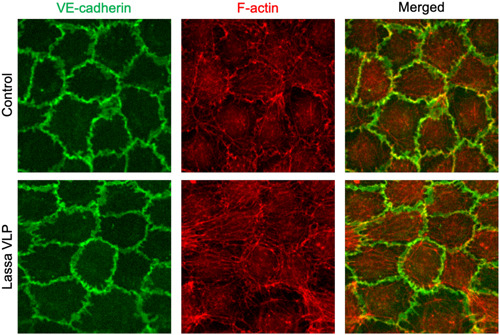
Microvascular dysfunction in the Lassa HS‐on‐a‐chip. Endothelial cells stained for VE‐cadherin (green) and F‐actin (red) after exposure to 50 μg/ml Lassa VLPs for 4 h. A relative increase in actin filament stress fiber formation was observed. HS, hemorrhagic shock; VLP, virus‐like particle [Color figure can be viewed at wileyonlinelibrary.com]

Immunostaining shows that treatment with Lassa VLPs did not result in an apparent rearrangement of VE‐cadherin, but instead altered the mechanics and physical interaction of endothelial cells (F‐actin), partially explaining the induction of permeability (Figure 3). Pearson's correlation coefficient was lower in microvessels exposed to Lassa VLPs (0.254 ± 0.04) than in the negative control (0.349 ± 0.06), showing an increase in stress fiber formation and endothelial cell activation (*p* < .01).

**Figure 4:**
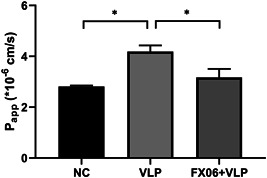
FX06 protects the vascular integrity in the Lassa HS chip platform. Microvessels (*n* = 5 per condition) exposed to Lassa VLP (10 μg/ml) were treated with FX06 (1 mg/ml) for 4 h, followed by permeability assay. Fibrin‐derived peptide FX06 is able to suppress the Lassa‐induced vascular integrity loss. FX06 treated group is not significantly different from the NC. HS, hemorrhagic shock; NC, negative control; VLP, virus‐like particle

We performed a small‐scale proof‐of‐concept study to demonstrate that the platform can be used for drug development. The effect of a potential drug, FX06, was tested by this platform, which previously proved promising for Ebola HS as shown in our previous paper (Junaid et al., 2020), but its therapeutic effect has never been tested for Lassa HS. Briefly, we compared vascular permeability of the microvessels exposed to Lassa VLP only with microvessels exposed to Lassa VLP and FX06 (F4 Pharma GmbH) simultaneously. To prevent the degradation of FX06 in the microvessels, 100 nM carboxypeptidase inhibitor (C0279; Sigma‐Aldrich) was added. Our data suggests that FX06 efficiently protects the vascular integrity in the Lassa HS‐on‐a‐chip platform (Figure 4).

The simple and robust Lassa HS‐on‐a‐chip described in this communication minimally mimics the Lassa disease‐associated vasculopathy. The platform allows to investigate the direct contribution of Lassa VLPs in the vascular permeability beyond the host immunity and the process of infection. Additionally, it can be adapted to develop new therapeutic compounds capable of delaying the effect of Lassa‐induced vascular leakage that either target endothelial cells or Lassa VLPs to block the interactions between them. Blood factors and immune cells can also be included to investigate their contributions to the integrity impairment in future. In addition, hiPSC derived cells can be used to generate individualized chips to probe interindividual variations in Lassa HS. Finally, this chip‐based platform can further be applied to combat other viral hemorrhagic diseases, such as Dengue, SARS‐CoV‐2 and many others.

## 2 MATERIALS AND METHODS

### 2.1 Cell culture

HUVECs were provided by Leiden University Medical Center. HUVECs were cultured in Endothelial Cell Basal Medium 2 (2% fetal calf serum, C‐22211; PromoCell) supplemented with Endothelial Cell Growth Medium 2 SupplementMix (C‐39216; PromoCell) and 1% pen‐strep under 5% CO_2_, 37°C incubator. HUVECs between Passages 3 and 4 were used in all experiments.

The OrganoPlate 2‐lane (9603‐400‐B; Mimetas) was used for microfluidic cell culture. 3D Culture Matrix Rat Collagen I (3447‐020‐01; Trevigen) neutralized with 10% 37 g/L Na_2_CO_3_ (S5761; Sigma) and 10% 1 M HEPES buffer (15630‐056; Gibco) to a final concentration of 4 mg/ml was dispensed into the ECM channels of the OrganoPlate, and incubated at 5% CO_2_, 37°C for 10 min to polymerize the collagen. For optical clarity and to prevent gel dehydration, 50 µl Hanks' balanced salt solution (HBSS, 14025050; Gibco) was added to the observation windows. Afterwards, 20 × 10^6^ cells/ml HUVECs were seeded into the gelatin‐coated microvascular channels and incubated for 1 h to allow for the formation of the microvessels. Subsequently, 100 μl cell culture medium was added to the microvascular channels. The device was then placed on an interval rocker platform with a 7° inclination and 8 min cycle time to allow continuous perfusion in the microvessels. Medium was refreshed after 24 h, and HUVECs were cultured for additional 3–4 days.

### 2.2 Vascular permeability assay

Microvascular channels were refreshed with 1, 10 and 50 μg/ml concentrations of Lassa VLPs (Lineage IV, LASVLP‐IV‐003; Zalgen Labs) diluted by Endothelial Cell Basal Medium 2 (0.2% serum) and incubated for 4 h. To measure vascular permeability, 20 µl HBSS was used to refresh the ECM channels. Then, the medium in the inlets and outlets of the microvascular channels were replaced with 40 and 30 µl of 125 µg/ml Alexa Fluor 555‐conjugated albumin (A34786; Invitrogen), respectively. Next, the device was transferred to a fluorescence microscope (Nikon Eclipse Ti) with an environmental chamber (5% CO_2_, 37°C) to take time‐lapse images.

For quantification purposes, we calculated the permeability coefficient by determining the fluorescence intensities in the microvascular (*I*_vessel_) and ECM (*I*_ECM_) channels of the captured images and normalizing them to each other at each time point. The apparent permeability (*P*_app_) was calculated as follows:

(1) $$P_{\mathrm{app}}\left(\cdot 10^{-6}\mathrm{cm}/s\right)=\frac{d\left(I_{\mathrm{ECM}}/I_{\mathrm{Vessel}}\right)}{\mathrm{dt}}\cdot \frac{V_{\mathrm{ECM}}}{A},$$

where *I*_ECM_/*I*_Vessel_ is ratio of intensity between the ECM and microvascular channel, *V*_ECM_ is the volume of the ECM channel (4.54 × 10^−4 ^cm^3^), A is the surface area of the vessel wall between the ECM and microvascular regions (1.21 × 10^−2^ cm^2^). The scatter plot was fitted with a linear trend line to determine the slope. This calculation showed the change in the intensity ratio inside the ECM channels as a function of time.

### 2.3 Immunohistochemistry

Cell culture medium was aspirated from the microvascular channel, where the vascular structure and cells were fixed with 4% paraformaldehyde (PFA) in HBSS for 10 min at room temperature. Permeabilization was performed for 2 min with 0.2% Triton X‐100 (T8787; Sigma‐Aldrich), then blocked with 5% bovine serum albumin (BSA, A9647; Sigma‐Aldrich) in HBSS for 30 min and incubated with the primary antibody solution overnight at 4°C. Mouse anti‐human CD144 (1:100, 555661; BD Pharmingen) was used as primary antibody, followed by 1 h incubation with Hoechst (1:2000, H3569; Invitrogen), Phalloidin (1:200, P1951; Sigma‐Aldrich), and Alexa Fluor 488‐conjugated goat antimouse secondary antibody (1:250, R37120; Invitrogen). Z‐stack images of the stained cells were taken by a high‐content confocal microscope (ImageXpress Micro Confocal; Molecular Devices). Quantification of Pearsons's correlation coefficient for the colocalization of VE‐cadherin and F‐actin was performed using ImageJ plugin Coloc2. Results are depicted as mean ± *SEM* (*n* = 7).

### 2.4 Statistical analysis

Statistical analyses were performed by GraphPad Prism (version 8.1.1). Outliers in the box plots were identified by IBM SPSS Statistics 25. The plotted data is the mean ± *SEM* of data from three or four independent replicates. *p* Value was determined using unpaired Student's *t *test and one‐way analysis of variance followed with Dunnett's multiple comparisons test. *p* Value < .05 was considered as statistical significance. In all figures, *p < .05, **p < .01, ***p < .001, and ****p < .0001.

## CONFLICT OF INTERESTS

The authors declare that there are no conflict of interests.

## AUTHOR CONTRIBUTIONS

Alireza Mashaghi conceived, designed, and supervised the research. Huaqi Tang and Yasmine Abouleila conducted the experiments. Huaqi Tang analyzed the data and wrote the first manuscript draft. All authors revised the manuscript and approved the final version.
